# Subthreshold membrane currents confer distinct tuning properties that enable neurons to encode the integral or derivative of their input

**DOI:** 10.3389/fncel.2014.00452

**Published:** 2015-01-09

**Authors:** Stéphanie Ratté, Milad Lankarany, Young-Ah Rho, Adam Patterson, Steven A. Prescott

**Affiliations:** ^1^Neurosciences and Mental Health, The Hospital for Sick ChildrenToronto, ON, Canada; ^2^Department of Physiology and Institute of Biomaterials and Biomedical Engineering, University of TorontoToronto, ON, Canada; ^3^Pittsburgh Center for Pain Research, University of PittsburghPittsburgh, PA, USA

**Keywords:** action potential, spike, neural coding, integrator, differentiator, coincidence detector

## Abstract

Neurons rely on action potentials, or spikes, to encode information. But spikes can encode different stimulus features in different neurons. We show here through simulations and experiments how neurons encode the integral or derivative of their input based on the distinct tuning properties conferred upon them by subthreshold currents. Slow-activating subthreshold inward (depolarizing) current mediates positive feedback control of subthreshold voltage, sustaining depolarization and allowing the neuron to spike on the basis of its integrated stimulus waveform. Slow-activating subthreshold outward (hyperpolarizing) current mediates negative feedback control of subthreshold voltage, truncating depolarization and forcing the neuron to spike on the basis of its differentiated stimulus waveform. Depending on its direction, slow-activating subthreshold current cooperates or competes with fast-activating inward current during spike initiation. This explanation predicts that sensitivity to the rate of change of stimulus intensity differs qualitatively between integrators and differentiators. This was confirmed experimentally in spinal sensory neurons that naturally behave as specialized integrators or differentiators. Predicted sensitivity to different stimulus features was confirmed by covariance analysis. Integration and differentiation, which are themselves inverse operations, are thus shown to be implemented by the slow feedback mediated by oppositely directed subthreshold currents expressed in different neurons.

## Introduction

Nearly all neurons use action potentials, or spikes, to transmit information. But that does not mean that all neurons generate spikes in the same way or that all spikes convey the same information. Tuning properties differ between neurons. Neurons are often said to operate as integrators or coincidence detectors based on the time interval over which they sum inputs (Abeles, 1982; König et al., 1996; Ratté et al., 2013). When temporally dispersed inputs cumulatively depolarize the neuron to threshold, integration time is long; on the other hand, when temporally coincident inputs rapidly depolarize the neuron to threshold, integration time is short. According to this explanation, operating mode depends on the temporal patterning of inputs (Segundo et al., 1963) and other input properties, like excitatory postsynaptic current (EPSC) kinetics, that affect temporal summation (König et al., 1996). However, certain neurons respond preferentially or even exclusively to certain types of input, implying that such neurons have a preferred operating mode because of their intrinsic properties (Lundstrom et al., 2009; Ratté et al., 2013; Gjorgjieva et al., 2014). The present study focuses on the relationship between intrinsic neuronal properties and operating mode, and specifically on how operating mode derives from the operation—integration or differentiation—performed on the input by key voltage-dependent currents.

Voltage-dependent currents active at subthreshold voltages affect excitatory postsynaptic potential (EPSP) kinetics (Fricker and Miles, 2000; Magee, 2000; Prescott and De Koninck, 2005; Gastrein et al., 2011; Remme and Rinzel, 2011), which in turn affect the temporal summation of inputs. But encoding is a two-step process insofar as input-driven depolarization must be converted into all-or-none spikes. This digital conversion also differs across neurons as evident from the diversity of spiking patterns observed when equivalent stimulation is applied to different neurons (Hodgkin, 1948; Llinás, 1988; Connors and Gutnick, 1990; Prescott et al., 2008a). Indeed, integration and coincidence detection are best suited to different conversion strategies: Integrators must spike repetitively at a rate proportional to their graded depolarization whereas coincidence detectors should spike reliably when abruptly depolarized (which is best achieved in the absence of repetitive spiking) (Gutkin et al., 2003; Prescott et al., 2006; Prescott and Sejnowski, 2008). A parsimonious explanation of encoding, including how tuning differs between integrators and coincidence detectors, must therefore address both analog processing and digital conversion.

We hypothesized that the same subthreshold-activating currents responsible for different spike initiation dynamics (Prescott et al., 2008a) also modulate subthreshold voltage trajectories, and that it is this inextricable combination of perithreshold effects that controls neuronal operating mode. Previously, we focused on explaining how three qualitatively distinct spiking patterns, each corresponding to one of Hodgkin's three classes of excitability (Hodgkin, 1948), arose from a continuum in underlying biophysical properties (Prescott et al., 2008a). Moving beyond spiking patterns, the present study sought to identify if seemingly independent response properties linked to distinct operating modes arise from common biophysical mechanisms. To this end, we constructed minimal computer models that reproduced the response properties observed in two types of spinal sensory neurons, one that naturally behaves as an integrator and the other as a coincidence detector. Inclusion of a slow-activating subthreshold inward or outward current was sufficient to reproduce the full constellation of response properties in integrators and coincidence detectors, respectively. Further analysis revealed how such currents coordinately regulate the subthreshold voltage trajectory and spike initiation dynamics. Key modeling predictions were experimentally confirmed, thus demonstrating that slow-activating subthreshold current, depending on its direction, enables the neuron to calculate (and encode) the integral or derivative of its input.

## Methods

### Slicing and electrophysiology

All experimental protocols were approved by the Hospital for Sick Children Animal Care Committee and University of Pittsburgh IACUC and have been described previously in detail (Prescott and De Koninck, 2002). Briefly, adult male Sprague Dawley rats were deeply anesthetized and perfused intracardially with ice-cold oxygenated (95% O_2_ and 5% CO_2_) sucrose-substituted artificial cerebrospinal fluid (S-ACSF) containing (in mM) 252 sucrose, 2.5 KCl, 2 CaCl_2_, 2 MgCl_2_, 10 glucose, 26 NaHCO_3_, 1.25 NaH_2_PO_4_, and 5 kynurenic acid; pH 7.35; 340–350 mOsm. The spinal cord was removed by hydraulic extrusion and sliced in the parasagittal plane at a thickness of 300 μm. Slices were stored in room temperature ACSF (126 mM NaCl instead of sucrose and without kynurenic acid; 300–310 mOsm) until recording. Slices were transferred to a recording chamber constantly perfused at ~2 ml/min ACSF at room temperature. Neurons were viewed with gradient contrast optics using a Zeiss AxioExaminer microscope and were recorded with whole cell patch clamp using an Axopatch 200B amplifier (Molecular Devices, Palo Alto, CA) and electrodes filled with (in mM) 135 KMeSO_4_, 5 KCl, 10 HEPES, and 2 MgCl_2_, 4 ATP (Sigma, St Louis, MO), 0.4 GTP (Sigma). Traces were low-passed filtered at 3-10 KHz and sampled at 10 KHz.

### Computational modeling

Our model is based on the Morris-Lecar model (Morris and Lecar, 1981; Rinzel and Ermentrout, 1989) and has been described in detail (Prescott et al., 2008a). All simulations, unless otherwise indicated, are based on the following equations:

(1) $$\begin{array}{l} CdV/dt=I_{\text{stim}}-g_{\text{leak}}(V-E_{\text{leak}})-{\bar{g}}_{\text{Na}}m_{\infty }(V)(V-E_{\text{Na}})\\ \;-{\bar{g}}_{\text{K},\text{dr}}w(V-E_{\text{K}})-{\bar{g}}_{\text{sub}}z(V-E_{\text{sub}})\\ \;-{\bar{g}}_{\text{adapt}}a(V-E_{\text{K}}) \end{array}$$

(2) $$dw/dt=\phi \frac{w_{\infty }(V)-w}{\tau_{\text{w}}(V)}$$

(3) $$dz/dt=\left(\frac{1}{1+e^{(\beta_{\text{z}}-V)/\gamma_{\text{z}}}}-z\right)/\tau_{\text{z}}$$

(4) $$da/dt=\left(\frac{1}{1+e^{(\beta_{\text{a}}-V)/\gamma_{\text{a}}}}-a\right)/\tau_{\text{a}}$$

(5) $$m_{\infty }(V)=0.5\left[1+\tanh\left(\frac{V-\beta_{\text{m}}}{\gamma_{\text{m}}}\right)\right]$$

(6) $$w_{\infty }(V)=0.5\left[1+\tanh\left(\frac{V-\beta_{\text{w}}}{\gamma_{\text{w}}}\right)\right]$$

(7) $$\tau_{\text{w}}(V)=1/\cosh\left(\frac{V-\beta_{\text{w}}}{2\cdot \beta_{\text{w}}}\right)$$

where *V* is voltage and *w, z*, and *a* control the time- and voltage-dependent activation of *g*_K,dr_, *g*_sub_, and *g*_adapt_, respectively; *g*_Na_ is assumed to activate instantaneously and *m* is therefore always at steady state. The following parameters are the same for integrators and differentiators: *C* = 2 μF/cm^2^; leak conductance *g*_leak_ = 2 mS/cm^2^, *E*_leak_ = −70 mV; sodium conductance *g*_Na_ = 20 mS/cm^2^, *E*_Na_ = 50 mV, β_m_ = −1.2 mV, γ_m_ = 18 mV; delayed rectifier potassium conductance *g*_K,dr_ = 20 mS/cm^2^, *E*_K_ = −100 mV, ϕ = 0.15, β_w_ = −10 mV, γ_w_ = 10 mV; AHP-type adaptation conductance *g*_adapt_ = 5 mS/cm^2^, β_a_ = 0 mV, γ_a_ = 5 mV, and τ_*a*_ = 20 ms. Voltage-dependency of the subthreshold conductance *g*_sub_ was equivalent between the integrator and differentiator: β_z_ = −40 mV and γ_z_ = 10 mV. For the integrator, *g*_sub_ = 0.7 mS/cm^2^, τ_*z*_ = 2 ms, *E*_sub_ = E_Na_. For the differentiator, *g*_sub_ = 1.5 mS/cm^2^, τ_z_ = 10 ms, and *E*_sub_ = *E*_K_. We have not modeled processes that change slowly on the timescale of a single spike (e.g., slow inactivation of sodium or potassium channels) because those ultra-slow processes are too slow to interact dynamically with the faster processes controlling spike initiation; accordingly, those processes can influence the interactions controlling spike initiation by modulating the effective density (or availability) of fast-activating channels, but they do not otherwise contribute to the relevant nonlinearities.

Simulations were run in XPP (Ermentrout, 2002) using the Euler method with a time step of 0.05 or 0.1 ms. Phase plane analysis was also conducted in XPP. For calculating nullclines at time *t*, all variables not associated with the nullcline were held constant at their value at time *t*. Time points are indicated in associated figures. Bifurcation analysis was conducted with AUTO using the XPP interface.

### Noise stimuli and spike-triggered analysis

For electrophysiological experiments, stimulation was applied by injecting current through the pipette. For simulations, equivalent stimulus waveforms were applied via *I*_stim_. This included a noisy waveform generated through an Ornstein-Uhlenbeck process (Uhlenbeck and Ornstein, 1930),

(8) $$d\zeta =-\frac{\zeta }{\tau_{\text{noise}}}dt+\sigma_{\text{noise}}N_{\tau }\xi \left(0,1\right)\sqrt{dt}$$

where ξ(0,1) is a random number drawn from a Gaussian distribution with average 0 and unit variance, and *N*_τ_ is a scaling factor $\sqrt{2/\tau_{\text{noise}}}$ so that ζ(*t*) has unit variance before scaling by σ_noise_. ξ is white noise while ζ is colored noise whose spectral properties are controlled by the autocorrelation time τ_noise_. The spike-triggered average stimulus (STA-*I*_stim_) was calculated by taking the average across the stimulus waveform preceding each spike elicited by colored noise input; the STA-*I*_stim_ calculated from the shuffled spike train was subtracted so that the final STA-*I*_stim_ reflects stimulus fluctuations around the average sustained input.

Covariance analysis was conducted following the methods and conventions described by Fairhall et al. (2006). Colored noise was applied to each model neuron as described above but the underlying white noise was used to calculate the STA and spike-triggered covariarance (STC) to avoid autocorrelations in the stimulus. Surrogate data were generated through the same shuffling process described above.

## Results

### Experimental characterization of integrator and differentiator traits

We began by contrasting the response properties of second-order sensory neurons in lamina I of the spinal dorsal horn, focusing on tonic- and single-spiking neurons because those neurons exemplify the traits of (and are henceforth referred to as) integrators and differentiators, respectively. Tonic- and single-spiking neurons constitute distinct cell types that together comprise the majority of lamina I neurons (Prescott and De Koninck, 2002). They most likely represent inhibitory and excitatory interneurons, respectively (Prescott and Ratté, 2012) but that distinction does not affect the present investigation into why each cell type behaves the way it does and nor was it the intent of this study to explore how cellular properties impact network function and pain processing. Instead, we focus here on the biophysical basis for cellular properties with the goal of identifying how key computational operations are biophysically implemented.

**Figure 1:**
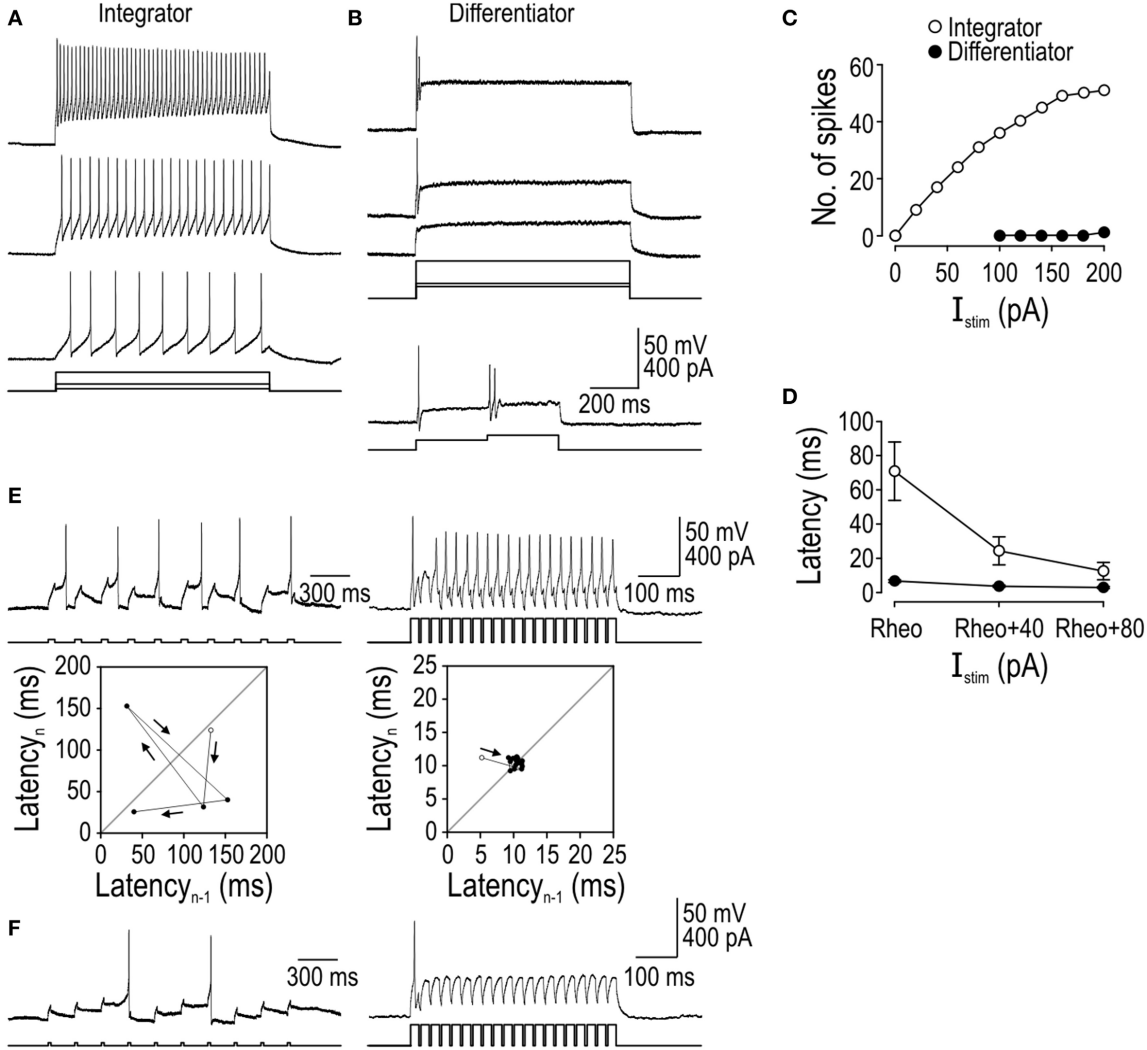
**Comparison of integrator and differentiator response properties in spinal neurons**. All sample traces are from a single tonic- or single-spiking cell that is representative of that cell type. **(A)** The integrator spikes repetitively during stimulation whereas the differentiator spikes only once or twice at the stimulus onset, even in response to strong stimulation as illustrated here with stimulation 2.5× rheobase (top right trace). **(B)** Differentiator can respond to subsequent increments in stimulus intensity. **(C)** Integrator encodes the stimulus intensity by modulating its firing rate (reported here as number of spikes per 900 ms-long stimulus) whereas the differentiator is incapable of such rate coding. **(D)** Mean latency from stimulus onset to first spike (±sem, *n* = 5 cells of each cell type) for stimulus intensities expressed relative to rheobase (i.e., minimum *I*_stim_ required to elicit spiking) identified independently for each neuron. **(E)** Trains of short, moderate-amplitude pulses elicit an irregular spike train in the integrator compared with a very regular spike train in the differentiator. Fidelity of spike timing is shown using return plots in which latency (from onset of the stimulus pulse) of one spike (*n*) is plotted against latency of the previous spike (*n*−1). Open circle indicates first data point. Spike latency in the integrator is sensitive to past spiking, unlike in the differentiator where spikes are time-locked to the underlying stimulus. **(F)** In response to stimulus pulses weaker than those in **(E)**, the integrator exhibits temporal summation whereas the differentiator does not, even at very short inter-pulse intervals.

Integrators spiked repetitively during prolonged current steps unlike differentiators, which spiked only at stimulus onset (Figure 1A). Differentiators could nonetheless respond to increments in stimulus intensity without requiring intervening hyperpolarization, as revealed by their response to biphasic current steps (Figure 1B). Because integrators fired repetitively, they could encode stimulus intensity by modulating their firing rate whereas differentiators could not (Figure 1C). Stimulus intensity also influenced spike latency in integrators whereas differentiators spiked at a consistently short latency (Figure 1D). Variability in spike latency had important consequences for how each cell type responded to stimulus trains: Differentiators responded with spikes time-locked to the individual pulses whereas integrators exhibited irregular spike timing (Figure 1E). Those effects are visualized here using return plots, which show latency (from stimulus onset) to spike *n* vs. latency of the previous spike *n-1*. The pattern observed for the integrator indicates that spike timing is influenced by previous spikes, which compromises phase-locking to the stimulus train; by comparison, the differentiator with its consistently short spike latency exhibited good phase-locking. When the cells received weaker stimulus pulses, the integrator exhibited temporal summation whereas the differentiator did not (Figure 1F). Absence of summation in the differentiator voltage response, despite a very short inter-pulse interval, is notable since temporal summation of the membrane potential could conceivably occur without evoking spikes purely because of the spike initiation process (e.g., because spikes must occur within a short latency of stimulus onset; see Figure 1E).

The temporal summation observed in tonic-spiking neurons is consistent with a long integration time, while its absence in single-spiking neurons is consistent with a short integration time. Beyond these differences in analog processing, tonic- and single-spiking neurons evidently differ in their digital conversion properties in ways beyond their namesake spiking patterns. Spiking pattern, spike latency/precision, and temporal summation represent a triad of response dimensions along which integrators and differentiators sit at opposite extremes, but it remains unanswered whether those properties derive from the same or different biophysical mechanisms.

### Reproduction of integrator and differentiator traits in a minimal model

**Figure 2:**
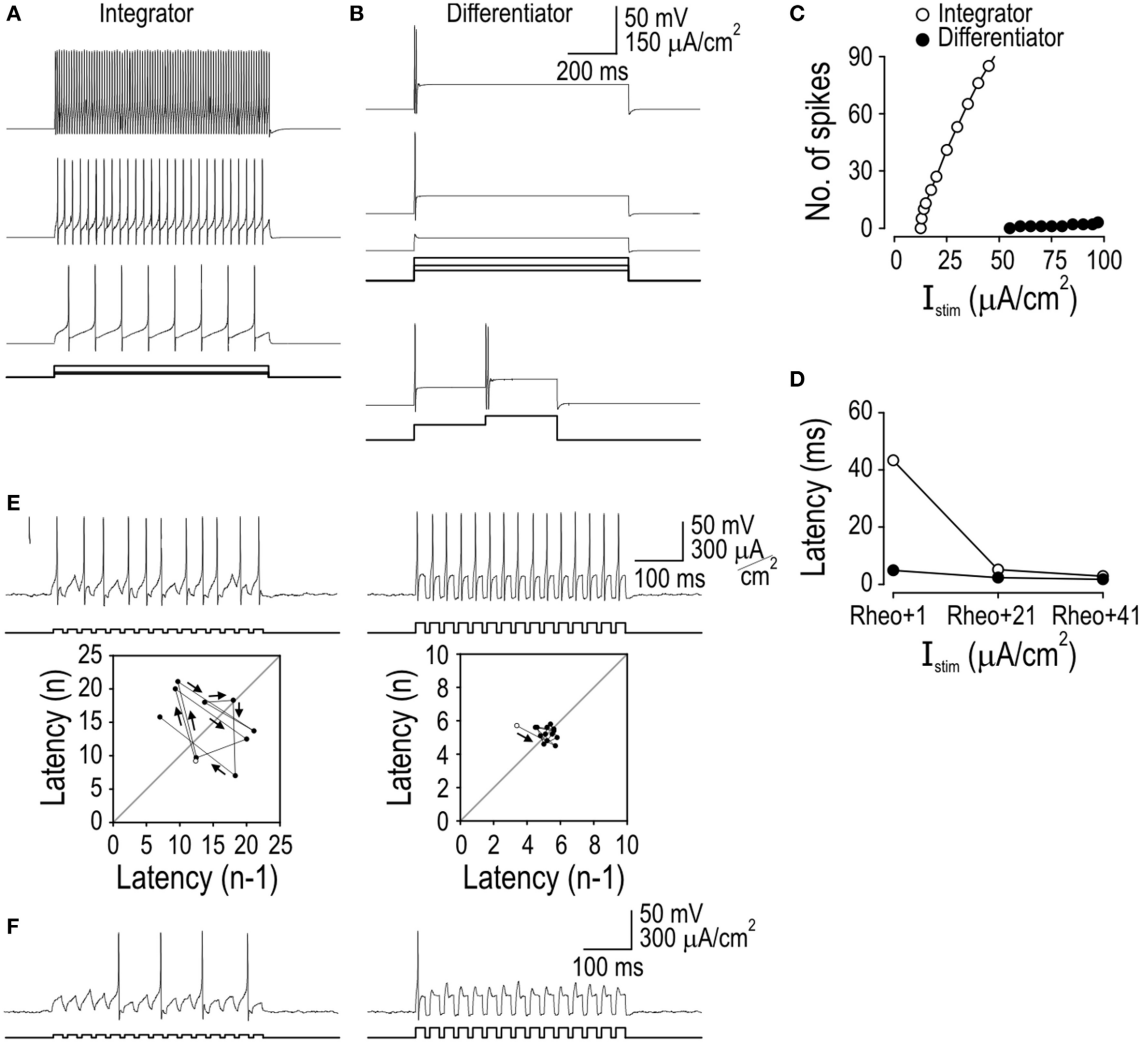
**Reproduction of integrator and differentiator response properties in a minimal model**. Addition of a subthreshold inward or outward current to a base model was sufficient to reproduce the responses to current steps **(A)**, responses to biphasic steps **(B)**, stimulus-response curves **(C)**, spike latency **(D)**, spike-timing precision **(E)**, and temporal summation **(F)** characteristic of integrators or differentiators, respectively. Small amplitude noise (τ_noise_ = 5 ms, σ_noise_ = 1 μA/cm^2^) was added to account for effects of intrinsic noise sources on spike timing.

To identify which biophysical mechanisms account for the response properties that distinguish integrators and differentiators, we sought (1) to reproduce each response pattern in the simplest possible computer model and (2) to convert that model between integrator and differentiator modes by varying as few parameters as possible. Starting from a “base” model, we added an inward or outward current to reproduce tonic- and single-spiking, respectively; the design of those subthreshold currents was based on previous work on spiking pattern (see Methods). The voltage-dependency of the added conductances were equivalent, thus isolating the reversal potential and maximal conductance as key differences between integrator and differentiator models. Maximal conductance was adjusted to give the desired spiking pattern (Figure 2A). We knew that spiking patterns could be reproduced in this way based on our previous work (Prescott et al., 2008a), but what is important here is that all *other* response properties were reproduced without further parameter changes (Figures 2B–F). This argues that the triad of response properties—spiking pattern, spike latency/precision, and temporal summation—originate from a common biophysical mechanism, namely the subthreshold current that was added to the base model.

As explained in more detail in the next section, the added current confers certain response properties by implementing delayed feedback. The voltage-dependency of that current dictates the voltage range in which that feedback operates; feedback must operate in a relatively narrow voltage range near threshold. The kinetics of that current dictates how the feedback responds to inputs with different kinetics; the feedback must operate primarily on slow inputs and therefore has a slow time constant between 2 and 10 ms which, although still quite fast, is slower than fast sodium channel activation and the fastest components of the input. Slow-activating subthreshold inward or outward current is sufficient to confer integrator or differentiator traits, respectively, but these data do not exclude neurons from differing in other ways [e.g., tonic- and single-spiking neurons differ in their dendritic morphology (Prescott and De Koninck, 2002) and in the degree of spike height accommodation] but any relationship with operating mode must be treated as correlative until proven otherwise. That said, other factors could, in theory at least, contribute to integrator or differentiator traits (see below). With respect to these spinal neurons, the subthreshold currents identified here as conveying integrator and differentiator traits are known to exist in tonic- and single-spiking neurons, respectively, are necessary for their associated spiking patterns, and have been implicated in shaping EPSP kinetics (Prescott and De Koninck, 2005; Prescott et al., 2008a). Specifically, integrators express a persistent sodium and calcium current whereas differentiators express a K_v_1-type potassium current.

### Differences in spike initiation dynamics

Next, we sought to explain how slow-activating subthreshold current (*I*_sub_) interacts with other currents to affect the spike initiation process. The nonlinear dynamics underlying spike initiation have been described in detail before (Rinzel and Ermentrout, 1989; Borisyuk and Rinzel, 2005; Izhikevich, 2007; Prescott et al., 2008a) but a description is provided here to explain the specific models developed in this study and to help establish experimentally testable predictions with respect to differences between integrators and differentiators. Moreover, our demonstrations emphasize the biophysical basis for nonlinear interactions and lead to interpretations that differ from those previously published (see also Discussion).

Briefly, bifurcation analysis describes how behavior of the system qualitatively changes (e.g., switches from quiescence to repetitive spiking) when a parameter is changed. Such changes reflect the nonlinear interaction between system variables. Those interactions can be visualized by plotting one variable against other variables to create a phase plane. Nullclines represent everywhere along the phase plane where a given variable does not change. Identifying how nullclines intersect and how those so-called fixed points change during a bifurcation can predict how system variables will evolve. For a more in-depth explanation of these sorts of analysis, see Izhikevich (2007).

**Figure 3:**
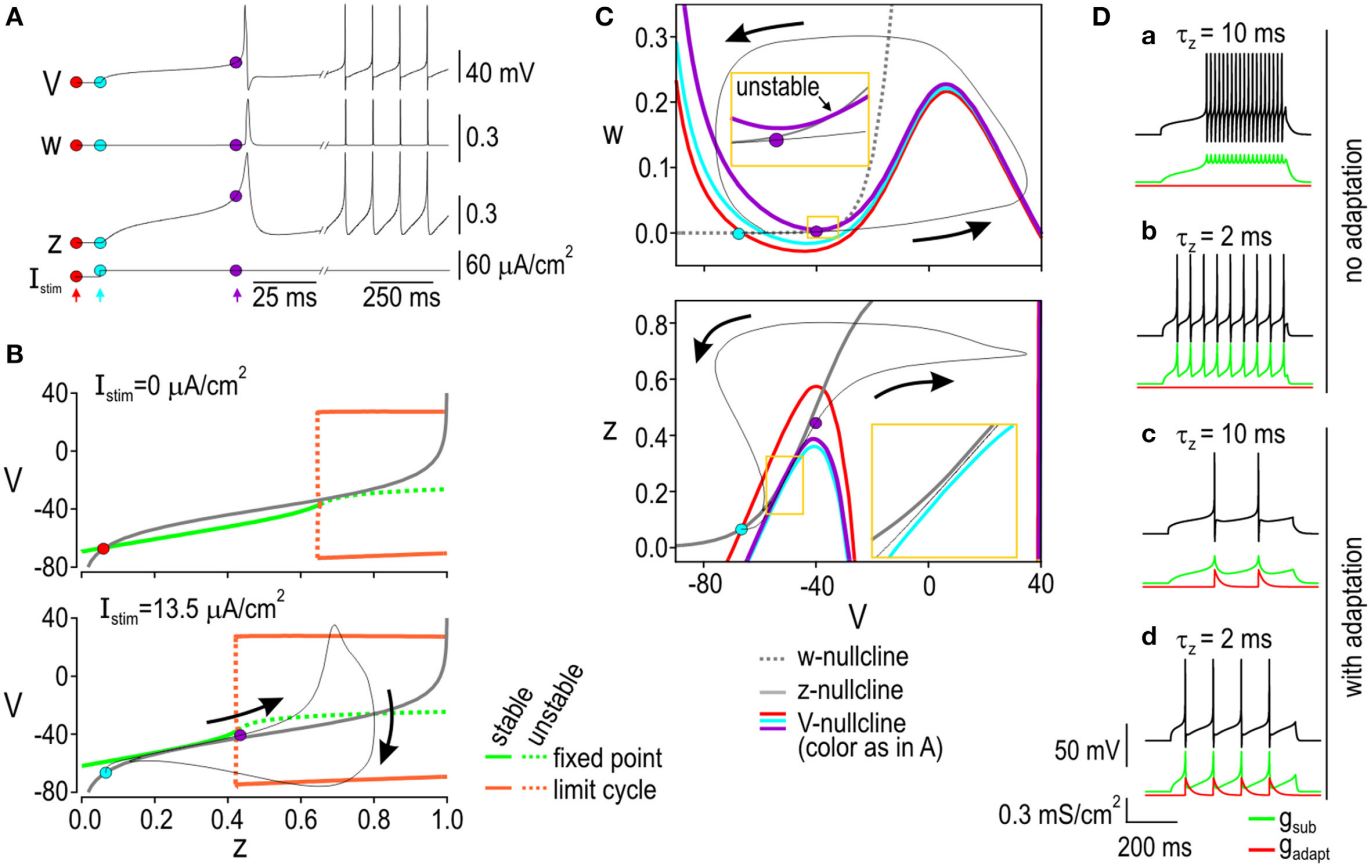
**Spike initiation dynamics in the integrator. (A)** Response of voltage (*V*), activation of *I*_K,dr_ (*w*) and activation of *I*_sub_ (*z*) to a stimulus current (*I*_stim_) plotted against time. Colored dots and arrows indicate time points illustrated in **(B,C)**. **(B)** Bifurcation diagrams at rest (top) and during stimulation (bottom) with *z* treated as a parameter and varied systematically from 0 to 1. Green curves show fixed point; orange curves show maximum and minimum of limit cycle. The bifurcation diagrams show that a Hopf bifurcation occurs (the fixed point becomes unstable and a stable limit cycle appears) if *z* is increased high enough. To predict how high *z* increases in the full model, the voltage-dependent activation curve for *I*_sub_ (gray) was overlaid on the bifurcation diagram; the response of the full model is shown in black. Without stimulation, the gray curve intersects the bifurcation diagram at a stable fixed point (red dot), which is where *z* remains in the full model. During stimulation, the fixed point is destroyed and the system moves from the blue dot toward the purple dot, after which a spike occurs. **(C)** Phase planes showing *V-w* interaction (top) and *V-z* interaction (bottom). Nullclines represent where a variable remains constant; *w*- and z-nullclines are shown in gray; *V*-nullclines vary with time and are shown in different colors corresponding to time points indicated in **(A)**. In the *V*-*w* phase-plane, stimulation shifts the *V*-nullcline upward (from red to blue position) but the intersection between the *V*- and *w*-nullclines is not destabilized until the *V*-nullcline is shifted further (to purple position) secondary to activation of *I*_sub_. Activation of *I*_sub_ can be understood from the *V*-*z* phase plane, where stimulation shifts the *V*-nullcline far enough downward that it no longer intersects the *z*-nullcline. Spike initiation in the integrator can therefore be described as a sequence of two bifurcations: a SNIC bifurcation in the *V-z* plane that facilitates a subsequent Hopf bifurcation in the *V-w* plane. Yellow boxes show enlarged view of critical regions. **(D)** Effects of subthreshold current activation time constant (τ_z_) and spike-dependent adaptation. **(a)** Although slow to start, spiking is very fast for τ_z_ = 2 ms because *g*_sub_ remains activated between spikes. Activation of *g*_sub_ is interrupted between spikes if τ_z_ is lengthened **(b)** and /or spike-dependent adaptation is included **(c,d)**. Adaptation also serves to increase the dynamic range.

Starting with the integrator, Figure 3A shows how variables *V, w*, and *z* evolve during a depolarizing step stimulus, where *w* and *z* control activation of *g*_K,dr_ and *g*_sub_, respectively. To quantify the impact of subthreshold inward current, we conducted bifurcation analysis by systematically increasing the activation of that current (controlled by *z*) rather than letting *z* evolve freely as a variable. Notably, this bifurcation analysis differs from the more typical analysis in which stimulating current *I*_stim_ is varied. As *z* was systematically increased, the stable fixed point destabilized through a Hopf bifurcation and was replaced with a stable limit cycle; the value of *z* at which this occurs depended on stimulus intensity (Figure 3B). To predict behavior of the full model (i.e., with *z* treated as a variable), we projected the voltage-dependent activation curve for *I*_sub_ onto the bifurcation diagram. Prior to stimulation (Figure 3B top), the activation curve intersected the bifurcation diagram in a region corresponding to a stable fixed point, which accurately predicts that the full model will rest at a subthreshold voltage with minimal activation of *I*_sub_. During stimulation (Figure 3B bottom), the bifurcation diagram is shifted such that it no longer intersects the activation curve at a stable fixed point; under those conditions, the full model spikes repetitively because of unrestricted activation of *I*_sub_. This is further illustrated by phase-plane analysis. In the *V-w* phase-plane (Figure 3C top), stimulation shifts the *V*-nullcline upwards (from red to blue position) but this is not enough to destabilize the intersection between the *V*- and *w*-nullclines; it is only after the *V*-nullcline is shifted further (to the purple position) by the contribution of *I*_sub_ that a Hopf bifurcation occurs. Activation of *I*_sub_ can be understood from the *V-z* phase-plane (Figure 3C bottom), where stimulation is sufficient to shift the *V*-nullcline far enough downwards that its stable intersection with the *z*-nullcline is destroyed through a saddle-node on invariant circle (SNIC) bifurcation. That bifurcation un-restricts the activation of inward *I*_sub_, leading to positive feedback depolarization and to the Hopf bifurcation in the *V-w* subsystem, whereupon repetitive spiking ensues.

An adaptation current was included in the integrator model to help reproduce the experimental data. Figure 3D shows the effects of the adaptation current and of varying the activation time constant for the subthreshold current, τ_z_. If τ_z_ is long, the first spike is slow to occur but the firing rate thereafter is high because the positive feedback is slow to initially activate but then remains activated between spikes (Figure 3Da). This is inconsistent with experimental data (Figure 1A) and is corrected by shortening τ_z_ so that the positive feedback (i.e. subthreshold inward current) deactivates between spikes (Figure 3Db). However, positive feedback results in even weakly suprathreshold stimuli causing high firing rates, thus causing the dynamic range to be narrow. The dynamic range is broadened by inclusion of a spike-dependent adaptation that activates abruptly with each spikes and decays slowly, thus intermittently interrupting the positive feedback process (Figures 3Dc,d). The prominent afterhyperpolarization (AHP) observed in tonic-spiking neurons is consistent with spike-dependent adaptation in this cell type (Prescott and De Koninck, 2002). Equivalent adaption was included in the differentiator model for sake of comparison but it had no notable effects.

**Figure 4:**
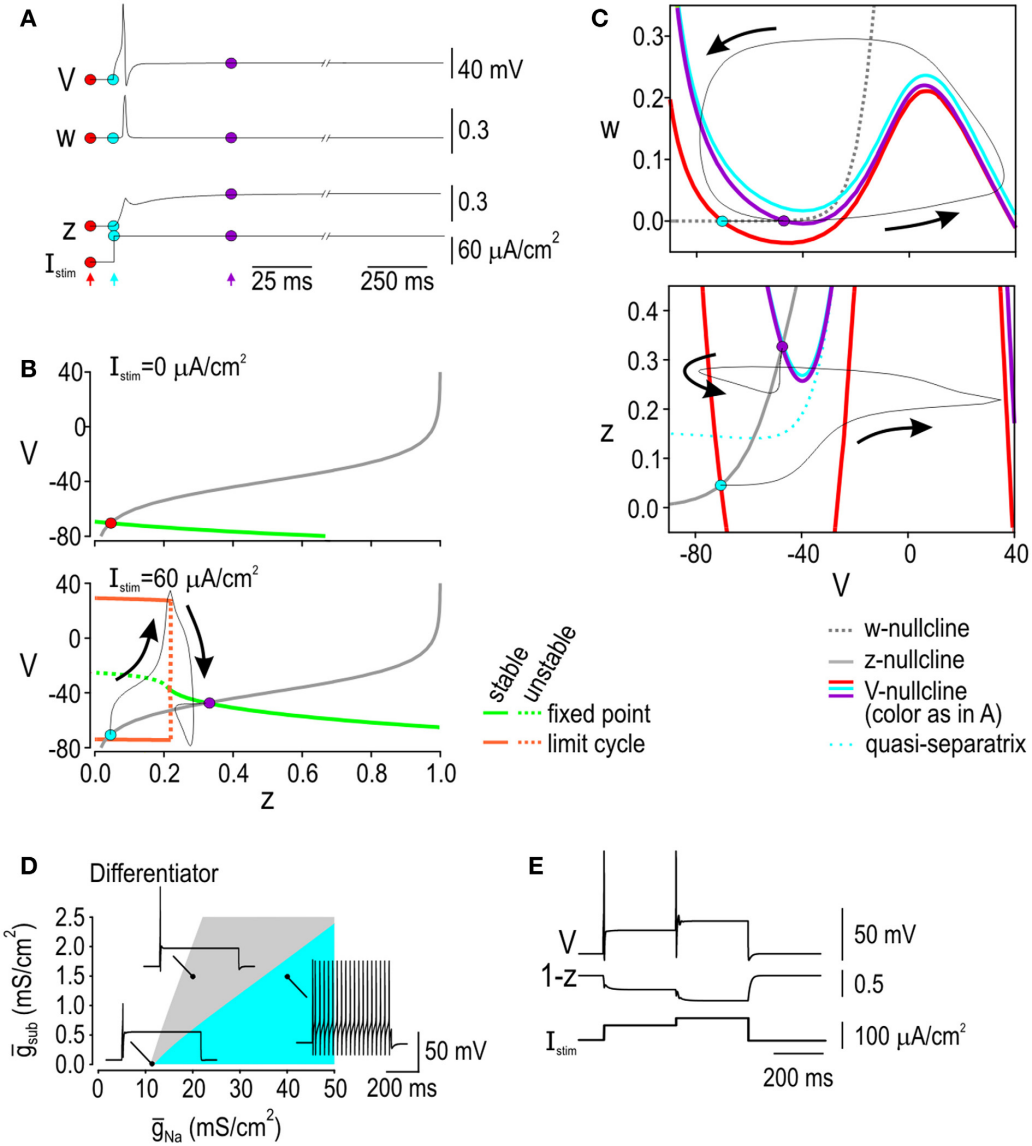
**Spike initiation dynamics in the differentiator. (A)** Variables plotted against time, like in Figure 3A. Colored dots and arrows indicate time points illustrated in **(B,C)**. **(B)** Bifurcation diagrams at rest (top) and during stimulation (bottom). Stimulation is required for a limit cycle to exist, but that limit cycle is lost if *z* is increased high enough. To predict how high *z* would increase in the full model, the voltage-dependent activation curve for *I*_sub_ (gray) was overlaid on the bifurcation diagram; the response of the full model is shown in black. Without stimulation, the gray curve intersects the bifurcation diagram at a stable fixed point (red dot). During stimulation, the gray curve intersects the bifurcation at a stable fixed point, but the system must move from the blue dot to the purple dot, and can produce a spike along the way. **(C)** Phase planes showing *V-w* interaction (top) and *V-z* interaction (bottom). In the *V*-*w* phase-plane, stimulation shifts the *V*-nullcline upward (from red to blue position), destabilizing the intersection between the *V*- and *w*-nullclines in the process, but that intersection is re-stabilized when activation of *I*_sub_ shifts the *V*-nullcline downward. Activation of *I*_sub_ can be understood from the *V*-*z* phase plane, where stimulation shifts the *V*-nullcline upward, although its intersection with the *z*-nullcline remains stable. A single spike is generated because the quasi-separatrix shifts above the blue dot, meaning the trajectory must follow an indirect route to the purple dot. In other words, crossing the quasi-separatrix in the *V-z* subsystem corresponds to the fixed point in the *V-w* subsystem remaining unstable long enough for a spike to be produced. **(D)** Effects of ultra-slow sodium channel inactivation modeled as a static change in sodium channel density (*g*_Na_). Blue and gray region show parameter combinations giving repetitive or onset-only spiking, respectively. Graph shows how density of subthreshold outward current (*g*_sub_) needed for onset-only spiking depends on *g*_Na_. Data are shown for *I*_stim_ = 60 μA/cm^2^; regions shift depending on *I*_stim_ (not shown). Onset-only spiking can be produced in the absence of subthreshold outward current if *g*_Na_ is sufficiently low but only for a narrow parameter range (i.e., gray region gets narrower as *g*_sub_ is reduced) whereas onset-only spiking is produced across a broad range of *g*_Na_ when outward *g*_sub_ is higher. **(E)** Effects of slow sodium channel inactivation modeled dynamically by having variable *z* control inactivation (see Results). Sample response to biphasic stimulus steps reproduces the experimental data in Figure 1B. Since changes in *z* are described by the same differential equation as in the original model (see Equation 3), its interactions with other variables are the same as described in panel C for the original differentiator model.

Spike initiation dynamics in the differentiator are completely different from those in the integrator insofar as differentiator spiking must occur despite subthreshold outward current rather than with the assistance of subthreshold inward current. Figure 4A shows how variables *V, w*, and *z* evolve during a depolarizing step stimulus. Bifurcation analysis using *z* as the bifurcation parameter shows that there is no limit cycle in the absence of stimulation (Figure 4B top). With sufficiently strong stimulation, a stable limit cycle exists but only at low values of *z* (Figure 4B bottom), which implies that repetitive spiking is possible but will be prevented if *I*_sub_ activates strongly enough. Notably, the activation curve for *I*_sub_ intersects the bottom bifurcation diagram in a region corresponding to a stable fixed point. Simulations in the full model show that the model stabilizes at that intersection but not before producing a single spike. This suggests that if the differentiator is to spike, it must do so before *I*_sub_ becomes too strongly activated, which is consistent with results of phase-plane analysis. In the *V-w* plane (Figure 4C top), stimulation shifts the *V*-nullcline upwards (from red to blue position), which is sufficient to cause a Hopf bifurcation, but activation of *I*_sub_ shifts the *V*-nullcline downwards so that it once again intersects the *w*-nullcline at a stable fixed point. Activation of *I*_sub_ can be understood from the *V-z* nullcline (Figure 4C bottom). Stimulation shifts the *V*-nullcline upwards but its intersection with the *z*-nullcline remains stable; despite this, simulations in the full model show that the trajectory follows an indirect route from the originally positioned stable fixed point to the newly positioned stable fixed point. This circuitous trajectory can occur because *z* does not change instantaneously, thus allowing the system to escape transiently from a stable fixed point when that point moves too rapidly (e.g., during abrupt onset of a stimulus step). Whether the system escapes far enough from the fixed point to produce a spike is explained by whether the trajectory crosses a quasi-separatrix—a boundary separating flow on the phase plane. Accordingly, we refer to spike initiation through this mechanism as a quasi-separatrix-crossing (Prescott et al., 2008a).

According to the above description, spike initiation in the differentiator depends on the competition between slow-activating potassium current and fast-activating sodium current. It is, therefore, notable that sodium channel inactivation is more prominent among differentiators (see Figure 1) as this effectively reduces *g*_Na_ and biases the competition in favor of onset-only spiking (Prescott et al., 2008a; Rho and Prescott, 2012). If such inactivation were to occur slowly—so slow that inactivation is effectively constant during any one spike—then it can be modeled as a static change in *g*_Na_, whereas if inactivation occurs more rapidly, it must be modeled as a dynamic variable. To test the effects of slow inactivation, we co-varied *g*_Na_ and *g*_sub_ in our differentiator model and confirmed that onset-only spiking can be produced without subthreshold outward current but only for a narrow range of fast sodium conductance; onset-only spiking was more robustly produced when the model contained strong subthreshold outward current (Figure 4D). To test the effects of faster inactivation, we altered Eqn 1 (see Methods) to produce a new current balance equation,

$$\begin{array}{l} C\frac{dV}{dt}=I_{\text{stim}}-g_{\text{leak}}\left(V-E_{\text{leak}}\right)\\ \;-{\bar{g}}_{\text{Na}}\;m_{\infty }\left(V\right)\left(\text{1}-z\right)\left(V-E_{\text{Na}}\right)\\ \;-{\bar{g}}_{\text{K,dr}}\;w\left(V-E_{\text{K}}\right)-{\bar{g}}_{\text{adapt}}\left(V-E_{\text{K}}\right) \end{array}$$

where *z* now controls inactivation of the fast sodium current. All other parameters were unchanged from the original differentiator model—*z* has the same kinetics and voltage-dependency as in the original differentiator model—but *g*_sub_ is absent. This “inactivation” model accurately reproduced the onset-only spiking pattern, including the response to biphasic stimulus steps (Figure 4E). The same competition between positive and negative feedback occurs as in our original differentiator model but is now implemented through fast activation and slower inactivation, respectively, of the fast sodium current. This mechanism may help confer differentiator traits but the fact that differentiators express K_v_1 channels and spike repetitively after blockade of those channels (Prescott et al., 2008a) argues that activation of subthreshold outward current is the dominant (and necessary) negative feedback mechanism in differentiators in lamina I of the spinal dorsal horn.

To summarize, the integrator spikes repetitively when a SNIC bifurcation enables positive feedback activation of subthreshold inward current whereas the differentiator spikes only if its quasi-separatrix is crossed, which corresponds to stimulus-induced depolarization outrunning the negative feedback mediated (predominantly) by subthreshold outward current. In both models, a Hopf bifurcation must also occur to initiate positive feedback activation of the fast-activating sodium current. According to this explanation, the spike initiation process comprises two threshold mechanisms. In the integrator, both thresholds (the Hopf bifurcation and the SNIC bifurcation) depend on stimulus intensity. In the differentiator, one threshold (the Hopf bifurcation) depends on stimulus intensity but the other (the quasi-separatrix crossing) depends on the rate of change of stimulus intensity. These differences, which have obvious implications for neuronal tuning (see below), ultimately boil down to whether the slow feedback process cooperates or competes with the fast positive feedback directly responsible for spiking.

### Differential responses to ramp stimuli

The threshold mechanisms described above predict differences in tuning that are pivotal to the distinction between integration and differentiation. If differentiator spiking occurs only when stimulus-driven depolarization outpaces the negative feedback implemented by outward current, then the rate of change of stimulus intensity will be a crucial factor in their decision to spike. The same is not predicted for integrators; in fact, the positive feedback implemented by subthreshold inward current ought to mitigate the intrinsic leakiness of the cell membrane and thereby encourage (rather than actively discourage) temporal summation.

**Figure 5:**
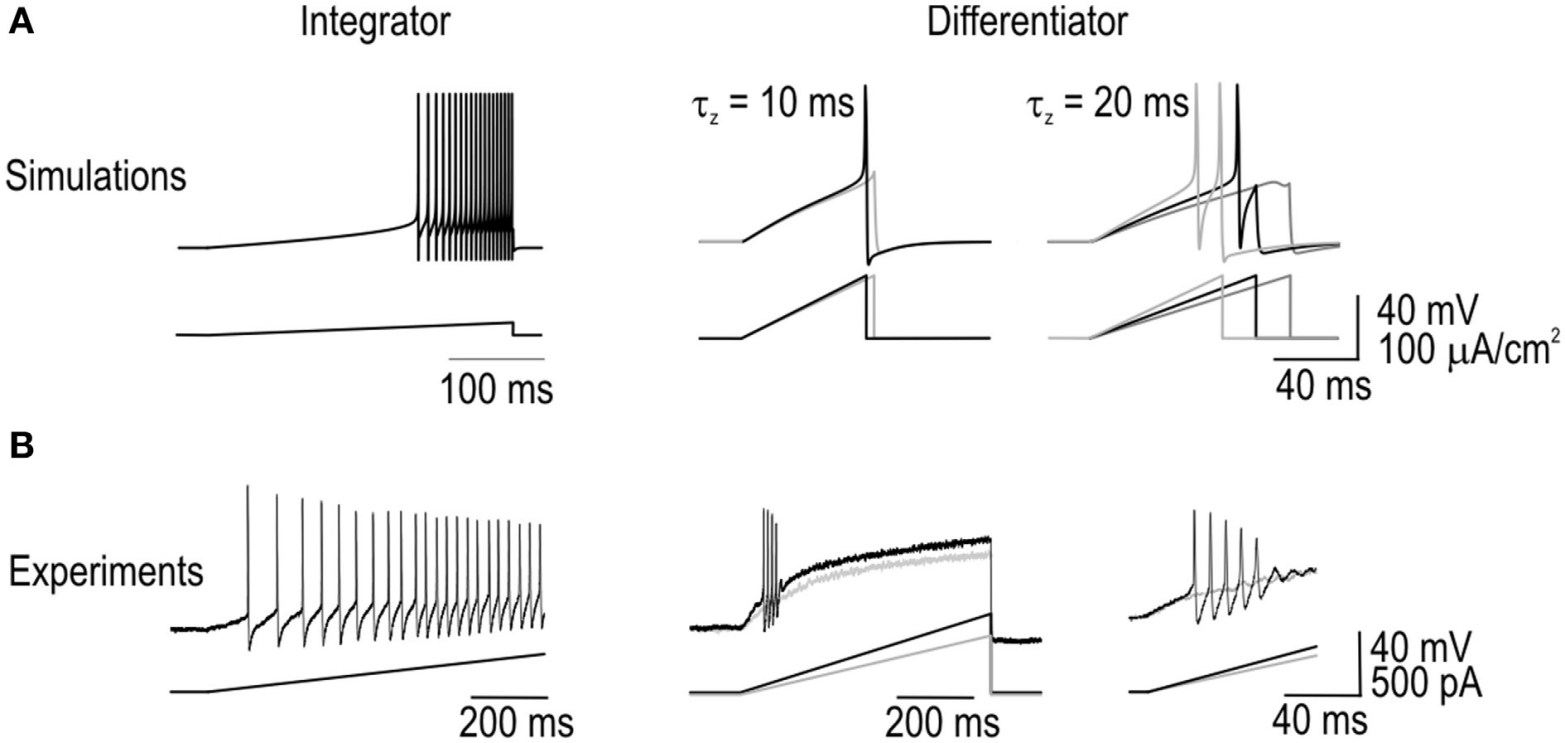
**Responses to ramp stimuli. (A)** Integrator model responded to extremely shallow ramps with repetitive spiking whereas differentiator models only responded to steep ramps. All ramps applied to differentiator models were capped below the intensity causing a Hopf bifurcation, thus ensuring that spikes occurred through a quasi-separatrix-crossing. Differentiator model was tested with the activation time constant for its subthreshold current τ_z_ = 10 ms (left) and 20 ms (right). As expected, slowing down the rate of negative feedback activation reduced the threshold stimulus slope. Differentiator model could spike more than once in response to supthreshold slopes. **(B)** Sample traces from each cell type confirming the model predictions. Sample responses from two differentiator neurons are shown. Experiments were repeated in three of each cell type.

Differential sensitivity to the rate of change of stimulus intensity was tested by applying ramp stimuli with different slopes. As expected, the integrator model responded to extremely shallow ramps with repetitive spiking whereas differentiator models responded only to ramps that exceeded a threshold slope (Figure 5A). Notably, the *V-z* subsystem of the differentiator model can experience a Hopf bifurcation at high stimulus intensities; accordingly, the peak intensity of each ramp was capped at a value below that intensity threshold. We hypothesized that slowing down the negative feedback (by lengthening τ_z_, the time constant controlling activation of subthreshold current) would reduce the threshold slope, which indeed it did (Figure 5A). Furthermore, fast ramps elicited >1 spike, as expected so long as the rate of stimulus-induced depolarization continues to exceed the rate at which negative feedback activates.

This ramp-driven repetitive spiking as well as the main prediction—that ramps must exceed a threshold slope to elicit differentiator spiking—were confirmed in 3 of 3 differentiator neurons tested whereas arbitrarily shallow ramps elicited repetitive spiking in 3 of 3 integrator neurons tested (Figure 5B). Notably, differentiator neurons were never seen to elicit >2 spikes in response to depolarizing steps with amplitude 2–3× rheobase, and so the observation of 4–5 spikes during a ramp is clearly not attributable to stimulus intensity. Based on the attenuation of spike amplitude, failure to continue spiking during the ramp is likely due to sodium channel inactivation.

### Differential processing of synaptic-like inputs

**Figure 6:**
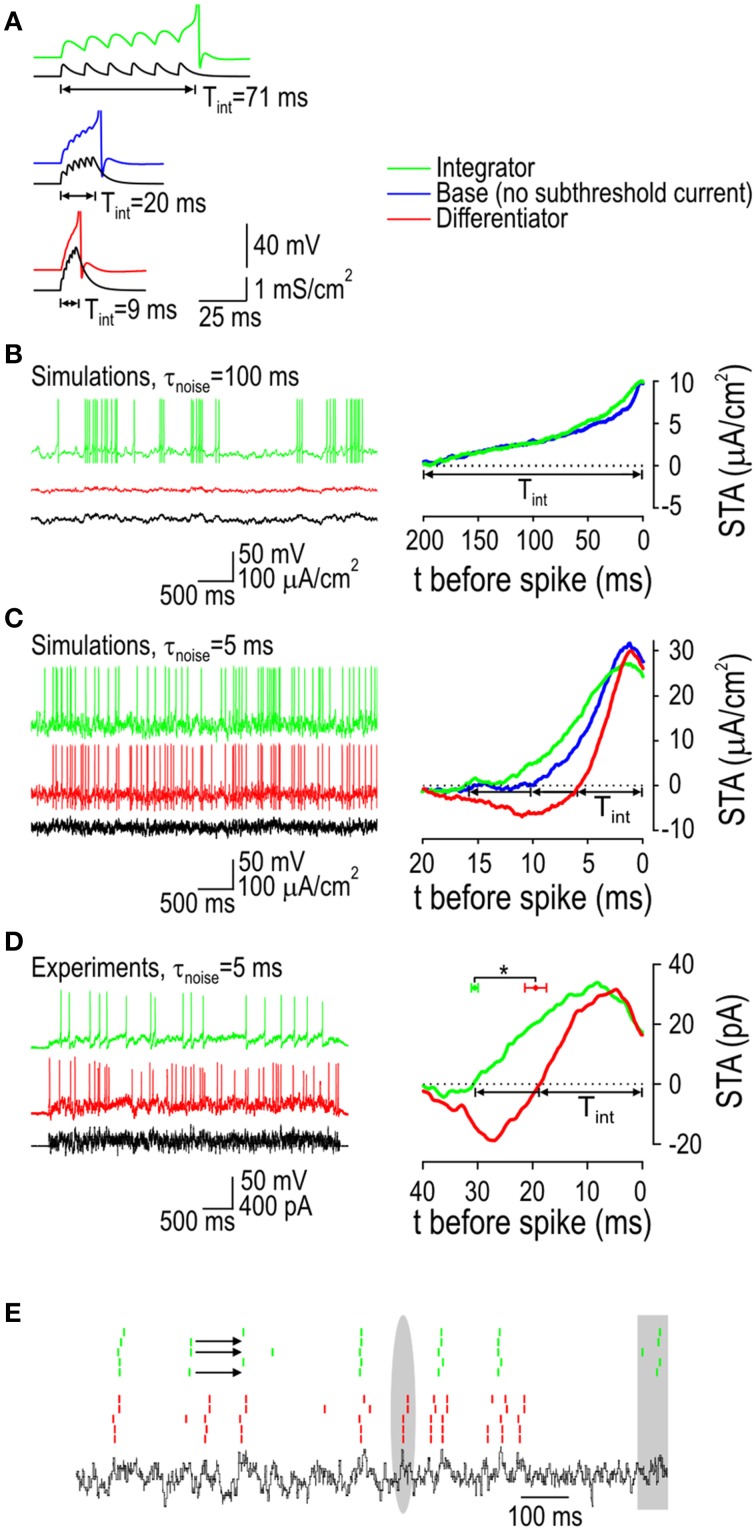
**Responses to synaptic-like stimuli. (A)** Response of model neurons to regularly spaced synaptic inputs modeled as *I*_syn_ = g_syn_(1-e^−t^/τ_rise_)e^−t^/τ_decay_(V-E_rev_), where *g*_syn_ = 0.7 mS/cm^2^, τ_rise_ = 0.5 ms, τ_decay_ = 5 ms, and *E*_rev_ = 0 mV. Inter-event interval was varied to find the minimum rate capable of eliciting a spike and integration time (*T*_int_) was estimated from the latency to spike. To simulate irregular synaptic-like input **(B–E)**, slow and fast fluctuating inputs were generated using an Ornstein-Uhlenbeck process with τ_noise_ = 100 ms or 5 ms. *T*_int_ was also measured as the duration of the positive phase of the spike-triggered average (STA) stimulus. **(B)** The integrator and base models responded to slow fluctuating input with *T*_int_ ≈ 200 ms but the differentiator did not, consistent with its need for rapid depolarization that is not achieved with this sort of input. With σ_noise_ = 10μA/cm^2^, average *I*_stim_ was 10 and 25 μA/cm^2^ for the integrator and base model, respectively, to get a firing rate of around 10 Hz. **(C)** All three models responded to fast fluctuating input, but whereas the integrator model exhibited a monophasic STA with *T*_int_ = 16.0 ms, the differentiator model exhibited a biphasic STA with *T_int_* = 5.8 ms. The base model was intermediate with *T*_int_ = 9.9 ms. With σ_noise_ = 10 μA/cm^2^, average *I*_stim_ was 3, 20, and 48 μA/cm^2^ for the integrator, base model, and differentiator, respectively, to get a firing rate of around 10 Hz. **(D)** Example STAs are shown for a typical integrator neuron (*T*_int_ = 30.8 ms) and a differentiator neuron (*T*_int_ = 18.7 ms) measured with fast-fluctuating input. Mean *T*_int_ ±SEM measured from three integrators (30.4 ± 0.4 ms) was significantly longer than that measured in three differentiators (19.4 ± 1.3 ms) (^*^p < 0.005, *t*-test). Average *I*_stim_ was adjusted for each cell to give firing between 5 and 10 Hz. **(E)** Rasters show spike times in response to “frozen noise” repeated five times in one of each cell type. The integrator and differentiator often responded to the same input events although there are clear instances (highlighted in gray) in which only one or the other responded. Arrows highlight examples (observed only in integrators) in which a response to preceding input event prevented the response to a subsequent input event that would have otherwise elicited a spike.

Sensitivity to stimulus intensity or to the rate of change of stimulus intensity impacts how synaptic input is processed. To explore this, we began by measuring the integration time (*T*_int_) based on excitatory postsynaptic currents (EPSCs) applied at regular intervals. Using a fixed-amplitude EPSC, we adjusted the interval to determine the maximum latency to spike, which was 8x less in the differentiator model compared with the integrator model, while the base model was intermediate (Figure 6A). To test responses to an irregular input comprising a broad mix of frequencies, we generated colored noise using an Ornstein-Uhlenbeck process and measured *T*_int_ by calculating the spike-triggered average stimulus (STA-*I*_stim_). Slowly fluctuating input (τ_noise_ = 100 ms) elicited vigorous spiking in the integrator and base models but not in the differentiator model (Figure 6B), which is consistent with the latter's need for rapid depolarization that is not achieved by this sort of input. In the integrator and base models, *T*_int_ was ~200 ms, consistent with the prolonged autocorrelation time of the input (Figure 6B). All models responded to faster fluctuating input (τ_noise_ = 5 ms), but whereas the integrator model exhibited a monophasic STA-*I*_stim_ with *T*_int_ = 16 ms, the differentiator model exhibited a biphasic STA-*I*_stim_ whose positive phase corresponds to *T*_int_ = 6 ms, and the base model was intermediate (Figure 6C). The exact integration time varies with stimulus parameters such as the average stimulus intensity, but the differentiator always had the shortest value and the integrator always had the longest. For data reported here, average stimulus intensity was adjusted to produce comparable firing rates in each cell type (see Figure 6 legend).

Based on the distinguishing STA-*I*_stim_ shapes revealed by testing with fast fluctuating input, we tested the same input experimentally. Like in simulations, integrator and differentiator neurons exhibited monophasic and biphasic STA-*I*_stim_s, respectively (Figure 6D). These data confirm that differentiators prefer more rapid depolarizing input than do integrators. Based on testing in three of each type of neuron, average *T*_int_ ± SEM was significantly longer in integrator neurons (30.4 ± 0.4 ms) than in differentiator neurons (19.4 ± 1.3 ms) (*p* < 0.005, *t*-test). Repeating the same fast fluctuating stimulus multiple times in the same neuron revealed some additional observations (Figure 6E). First, the integrator and differentiator neurons each tended to respond reliably across trials but the two types of neurons did not necessarily respond to the same input fluctuations. Also, the integrator neuron exhibited a pattern wherein responding to an earlier fluctuation could prevent it from responding to a subsequent fluctuation, which is reminiscent of data in Figures 1E, 2E.

**Figure 7:**
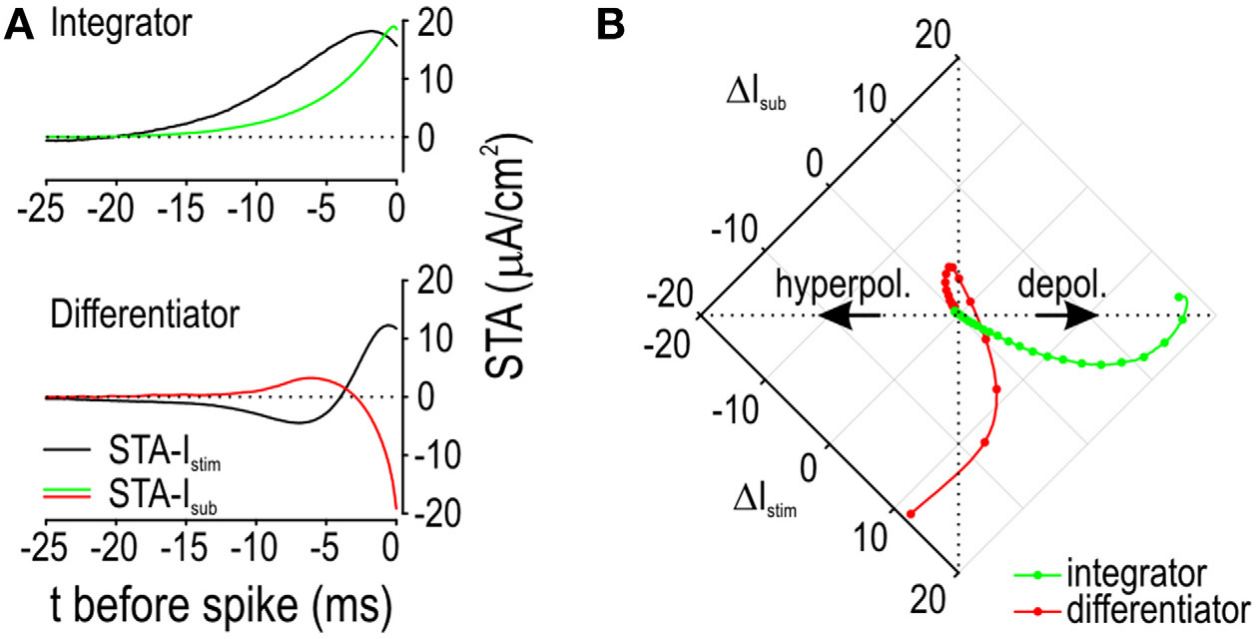
**Feature selectivity depends on how *I*_stim_ interacts with *I*_sub_. (A)** Spike-triggered averages were calculated for the stimulus (STA-*I*_stim_) and for the subthreshold current that was activated (STA-*I*_sub_). Depolarizing current is shown as an upward (positive) deflection. Average *I*_stim_ was 5 μA/cm^2^ and 80 μA/cm^2^ for the integrator and differentiator, respectively; σ_noise_ was 10 μA/cm^2^ for both. **(B)** Same data as in A but now with *I*_stim_ and *I*_sub_ plotted relative to one another rather than against time. Dots are plotted at 1 ms intervals to illustrate the rate at which each variable changes, where wide spacing represents rapid change. For the integrator, *I*_stim_ causes relatively slow depolarization, thus giving inward *I*_sub_ time to activate, which results in counterclockwise bending of the trajectory. Counterclockwise bending reflects cooperativity between *I*_stim_ and inward *I*_sub_, which increases the capacity of slow stimulus fluctuations to drive suprathreshold depolarization. For the differentiator, the initial slow reduction in *I*_stim_ causes deactivation of outward *I*_sub_, thus producing a net inward current. The subsequent rapid increase in *I*_stim_ drives depolarization while minimizing the time available for outward *I*_sub_ to activate before the spike is initiated. Clockwise bending of this trajectory reflects competition between *I*_stim_ and outward *I*_sub_, which ensures that only fast stimulus fluctuations drive suprathreshold depolarization.

We returned to the model to compare the STA-*I*_stim_ against activation of *I*_sub_, which we plot here as the spike-triggered average subthreshold current (STA-*I*_sub_). We reasoned that the positive phase of the differentiator STA-*I*_stim_ is short because this minimizes activation of outward *I*_sub_ whereas the positive phase of the integrator STA-*I*_stim_ is relatively long because this capitalizes on activation of inward *I*_sub_. Comparing the STA-*I*_sub_ for integrators and differentiators confirmed this predicted pattern (Figure 7A). Since the change in voltage depends on the sum of *I*_stim_ and *I*_sub_ (along with other transmembrane currents), we plotted STA-*I*_stim_ against STA-*I*_sub_ (Figure 7B). Dots are plotted at 1 ms-intervals to help visualize the rate of change. The integrator trajectory curls counterclockwise, confirming that slow depolarization driven by *I*_stim_ causes activation of inward *I*_sub_ so that by the time the spike occurs (at the end of the trajectory), *I*_stim_ and *I*_sub_ contribute equally to depolarization. On the other hand, the differentiator trajectory spirals in a clockwise direction, revealing how slow initial hyperpolarization by *I*_stim_ causes a net inward current by deactivating outward *I*_sub_; this is followed by rapid depolarization such that outward *I*_sub_ exceeds its baseline activation only 3 ms before the spike. If depolarization was slower, *I*_sub_ would activate more strongly, resulting in a tighter spiral, less depolarization, and no spike. A voltage threshold corresponds to a vertical boundary on Figure 7B. One should appreciate that that the differentiator trajectory might not cross that boundary (even if *I*_stim_ is very strong) because activation of outward *I*_sub_ causes the trajectory to veer away. In contrast, activation of inward *I*_sub_ causes the trajectory to veer toward such a boundary, thus increasing the likelihood that the voltage threshold is reached. These results emphasize how the selectivity for different stimulus features (i.e., the capacity for different stimulus waveforms to evoke a spike) depends on how the stimulus interacts with the subthrehsold current.

### Spike-triggered covariance analysis of sensitivity to stimulus features

As demonstrated by the responses to ramp stimulation (Figure 5), differentiator spiking is sensitive to the rate of change of stimulus intensity and to stimulus intensity itself. In that respect, neither our conductance-based differentiator model nor our differentiator neurons are *pure* differentiators. We hypothesized that this joint sensitivity to the stimulus intensity and its rate of change arises from the two requirements for differentiator spike initiation: A Hopf bifurcation in the *V-w* subsystem depends on stimulus intensity while the quasi-separatrix-crossing in the *V-z* subsystem depends on the rate of change of stimulus intensity (see Figure 4). Both thresholds must be satisfied. By comparison, integrator spike initiation depends on a Hopf bifurcation in the *V-w* subsystem *or* a SNIC bifurcation in the *V-z* subsystem (see Figure 3). The SNIC bifurcation is inconsequential if stimulus intensity is sufficient to cause the Hopf bifurcation on its own; on the other hand, the Hopf bifurcation becomes inevitable once the SNIC bifurcation occurs so long as stimulation is not abruptly discontinued. In other words, the two intensity thresholds amount to a single intensity threshold whose precise value can vary, unlike the dual-threshold predicted for differentiators. To summarize, we reasoned that sensitivity to different stimulus features, namely stimulus intensity and its derivative, is a direct consequence of the two dynamical processes comprising the spike initiation process.

**Figure 8:**
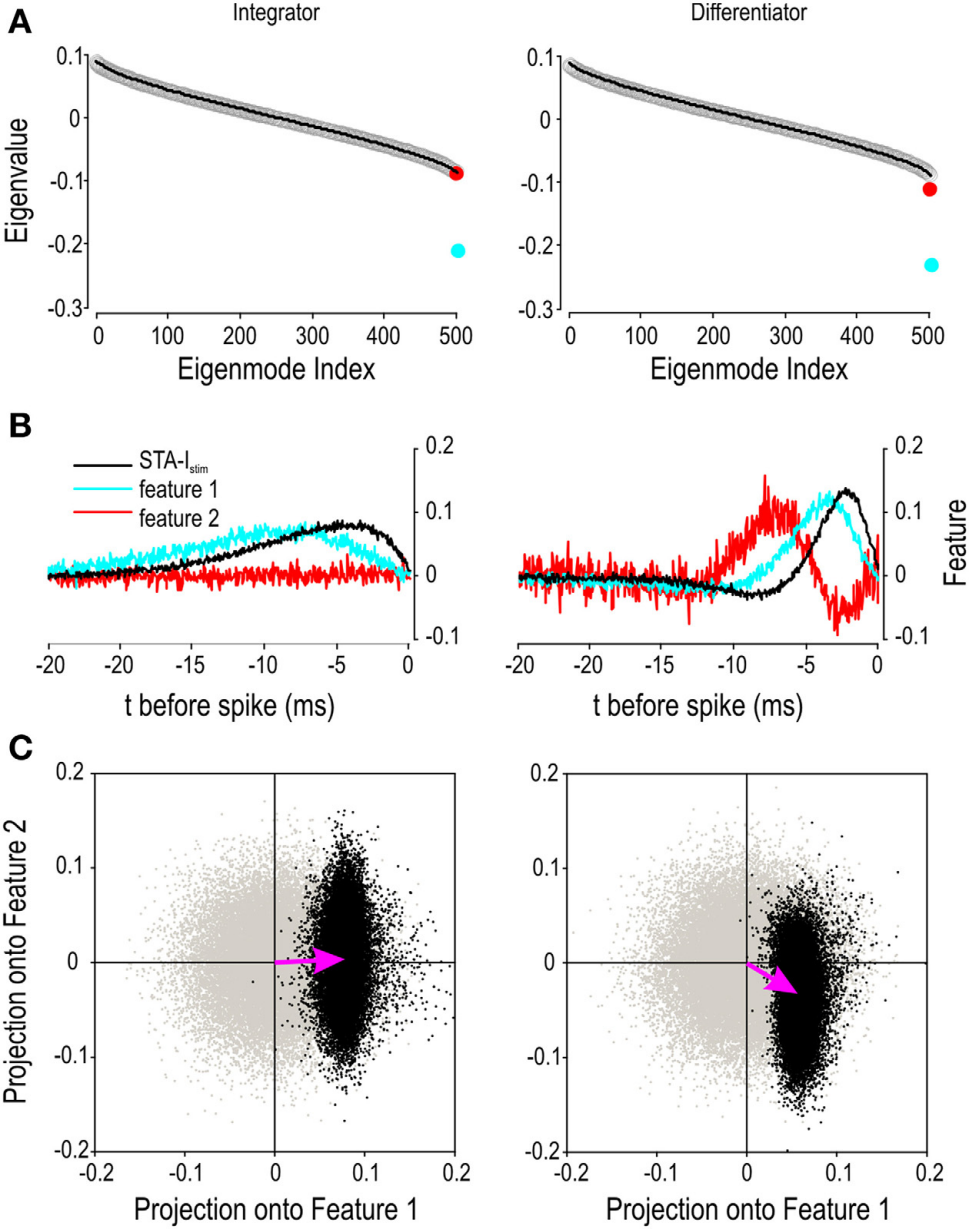
**Differences in encoding properties revealed by co-variance analysis**. Stimulus parameters were as reported in Figure 7 but the underlying white noise was used for all analysis. **(A)** Rank-ordered eigenvalues are shown for spike-triggered data (circles) and for surrogate data (black line) based on a spike-triggered stimulus ensemble found after shuffling the spike times. Ten surrogate data sets were generated by re-shuffling the spike times. The two smallest eigenmodes deviated significantly from the surrogate data (*p* < 0.01; one-sample *t*-tests) for the differentiator whereas only one eigenmode deviated significantly in the case of the integrator. **(B)** STA-*I*_stim_ and the eigenvectors corresponding to the two smallest eigenmodes, which are designated *feature 1* and *2*. **(C)** When spike-triggered stimuli are projected onto features 1 and 2, they form a cloud (black points) that can be compared against surrogate data projected onto the same two features (gray points). Pink arrows, which correspond to the STA-*I*_stim_, show the difference in the center of mass of each cloud. The dimensions of the black cloud relative to the gray cloud reveal the stimulus features about which a spike conveys information. These data argue that integrators encode information about a single stimulus feature, namely stimulus intensity, whereas differentiators encode information about both stimulus intensity and its rate of change.

To investigate this further, we conducted spike-triggered co-variance analysis to identify the stimulus features to which each spike initiation mechanism is sensitive (see Methods). As explained by Fairhall et al. (2006), this analysis identifies directions in stimulus space along which variance differs relative to the prior stimulus distribution; the eigenvalue of each direction reveals the stimulus features (i.e., directions) to which the neuron is sensitive. Spike-triggered covariance revealed only one significant eigenvalue for the integrator model whereas two significant eigenvalues were found for the differentiator model (Figure 8A). For both models, the eigenvector for the smallest eigenvalue (identified as *feature 1*) resembles the STA for that cell type (Figure 8B). For the differentiator, the eigenvector for the second smallest eigenvalue (*feature 2*) resembles the derivative of feature 1 but is unstructured in the case of the integrator (Figure 8B). Next, we projected the spike-triggered stimulus ensemble onto the two features (Figure 8C). The spike-triggered stimuli form a distinct cloud that can be compared to the prior. The STA-*I*_stim_ describes how the mean of this cloud is offset relative to the prior while compression of the cloud reflects the information provided by each feature. For the integrator, STA-*I*_stim_ points horizontally and only feature 1 provides information. For the differentiator, STA-*I*_stim_ points diagonally and both features 1 and 2 provide information, thus confirming our prediction. Applying the same sort of analysis to experimental data recorded from differentiators in the auditory brainstem, Slee et al. (2005) found similar results, specifically that blockade of the low-threshold potassium current changed the projection onto the second feature (and the spiking predicted therefrom) although the second feature was still evident.

## Discussion

Using a combination of simulations and experiments, we have explored the biophysical mechanisms whereby neurons compute the integral or derivative of their input. We found that integrators and differentiators differ in multiple response properties—temporal summation, spike latency/precision, and spiking pattern—yet all of those differences have a common biophysical explanation, namely the direction of slow-activating subthreshold current. The direction of that current dictates whether the slow feedback on subthrehsold voltage is positive or negative. This, in turn, dictates whether temporal summation is encouraged or discouraged and it also leads to fundamentally different thresholding mechanisms based on how slow-activating subthreshold current interacts with the fast-activating positive feedback responsible for spiking.

In integrators, subthreshold *inward* current creates a positive feedback loop that sustains depolarization and interacts cooperatively with fast-activating sodium current to produce repetitive spiking at a rate proportional to stimulus intensity. The positive feedback loop is initiated when stimulus intensity exceeds the threshold required to produce a SNIC bifurcation in the interaction between voltage and the activation variable for the subthreshold current. This differs from the standard dynamical explanation of class 1 excitability, which explains slow repetitive spiking as originating from a SNIC bifurcation in the interaction between voltage and the recovery variable *w* (where *w* represents activation of the delayed rectifier potassium current) (Izhikevich, 2007; Prescott et al., 2008a; Prescott, 2015). In our integrator model, the effects of positive feedback are kept in check by the afterhyperpolarization (AHP) caused by spike-dependent adaptation. This amounts to a push-pull mechanism: Subthreshold inward current “pushes” membrane potential up (toward a second threshold defined by a Hopf bifurcation in the interaction between voltage and the recovery variable) while the AHP “pulls” membrane potential down. This leads to strong internal dynamics that depend on the AHP kinetics. Those internal dynamics have been shown previously to linearize the input-output curve (Ermentrout, 1998) and to cause noise shaping by introducing negative interspike interval correlations (Prescott and Sejnowski, 2008). Both effects selectively benefit the rate coding of slow signals with dense spiking (Chacron et al., 2004), and thus are relevant for integrators operating in a mean-driven or oscillatory regime. In the fluctuation-driven regime, which is characterized by sparse spikes each driven by separate stimulus fluctuations, the AHP may prevent bursting but is negligible from one fluctuation-driven spike to the next. Under those conditions, the positive feedback control of subthreshold voltage trajectory—the “push” mechanism—enhances temporal summation. Our noise-based analysis focused on the fluctuation-driven region.

By comparison, the subthreshold *outward* current expressed by differentiators creates a negative feedback loop that truncates depolarization and prevents repetitive spiking by interacting competitively with fast-activating sodium current. Equivalent negative feedback can in principle be achieved through slow inactivation of the fast-activating sodium current. Either mechanism might be said to mediate a form of *spike threshold accommodation*. An isolated spike is produced when stimulus-mediated depolarization causes a quasi-separatrix-crossing, which amounts to escaping the negative feedback by outrunning its activation. According to this explanation, spike initiation depends on the rate of change of stimulus intensity exceeding some threshold, and the threshold slope naturally depends on the activation kinetics of the subthreshold outward current. Lundstrom et al. (2009) have carefully demonstrated how the kinetics of that feedback tune neuronal sensitivity to fluctuating input. Our models predicted and experiments confirmed that differentiators spike repetitively as long as the rate of change of stimulus intensity exceeds the threshold slope. In theory at least, the rate of such spiking could encode the derivative of the stimulus. In practice, a rapidly depolarizing stimulus (such as that caused by a volley of synchronous inputs) is likely to be so brief that only one spike can occur during it. The timing of such spikes indicates when suprathreshold stimulus events occurred, while the rate of those spikes indicates the rate of suprathreshold stimulus events. What is most important is that differentiators, because of their selectivity for rapidly depolarizing input, respond selectively to synchronous inputs; furthermore, a set of such neurons receiving common input can respond with synchronous spikes, thus ensuring good synchrony-based coding (Hong et al., 2012; Ratté et al., 2013). Integrators can obviously respond to synchronous inputs, but a set of such neurons does not necessarily respond with good output synchrony. In effect, differentiators always operate in a fluctuation-driven regime and, moreover, respond selectively to fluctuations comprising relatively high frequencies because of the high-pass filtering implemented by the subthreshold outward current.

According to the explanations provided above (see also Figures 3, 4), spiking involves two thresholds. In the integrator, one threshold (the SNIC bifurcation) controls activation of the subthreshold inward current and the other (the Hopf bifurcation) controls activation of the fast-activating sodium current. In the differentiator, one threshold (the quasi-sepratrix crossing) describes whether stimulus-driven depolarization outruns activation of the subthreshold outward current while the other is the same Hopf bifurcation seen in the integrator. Having two thresholds begs the question of whether one threshold is dominant over the other, and if not, how the two combine. In the case of integrators, reaching the first threshold initiates a positive feedback process, which, given sufficient time, will insure that the second threshold is reached. Accordingly, the importance of the second threshold depends on the kinetics of the input relative to the activation kinetics of the subthreshold current; in other words, the second threshold becomes important only if the stimulus-induced depolarization is so short that the second threshold must be reached without the help of the subthreshold inward current; under those stimulus conditions, the neuron is not exploiting its integrating capacity in the first place. In the differentiator, both thresholds come into play insofar as depolarization must not only be rapid, but must also be sufficient in amplitude for a spike to occur. The first thresholding mechanism shapes the voltage trajectory before that voltage hits the second threshold, at which point a spike becomes truly inevitable.

The explanations of spike initiation provided here differ from previous descriptions (Rinzel and Ermentrout, 1989; Borisyuk and Rinzel, 2005; Izhikevich, 2007; Prescott et al., 2008a) and arguably provide deeper insights into the biophysical underpinnings of that process. This hinges on our use of a conductance-based model that is still low-dimensional yet slightly more complex than the simplest models upon which most nonlinear dynamical analysis has been applied. That said, our neuron models are still extremely simple compared with real neurons, which leads one to suspect that spatially complex neurons that express diverse ion channels with inhomogeneous densities may have multiple thresholding mechanisms that somehow combine to give a global threshold (Ashida et al., 2007; Brette, 2013). That said, the same neuron may combine thresholds in subtly different ways depending on the stimulus statistics, implying that the threshold may change, quantitatively at least, depending on the stimulus (Famulare and Fairhall, 2010) (see below). Qualitative differences exist between neurons, as exemplified here by our comparison of tonic- and single-spiking neurons in the spinal dorsal horn. Moreover, intrinsic neuronal properties are subject to modulation, which means the threshold can change (Prescott et al., 2008b; Hong et al., 2012).

The above discussion has emphasized neuronal properties and a neuron-centric definition of operating mode. Previous discussions of operating mode have most often emphasized the effects of stimulus properties (Mainen and Sejnowski, 1995; Nowak et al., 1997; Azouz and Gray, 2000, 2003; Rudolph and Destexhe, 2003; Axmacher and Miles, 2004). Both are important. This is illustrated by the responses to noisy stimuli (see Figure 6): The integrator responded to both slow- and fast-fluctuating input with STAs whose difference is entirely attributable to the difference in stimulus autocorrelation time; however, the integrator and differentiator neurons responded to the same fast-fluctuating stimulus with STAs whose difference is entirely attributable to difference in neuronal properties, namely their subthreshold current. Indeed, as illustrated in Figure 6E, the two cell types do not necessarily respond to the same stimulus events. This does not change the fact that integrators are sensitive to stimulus fluctuations; they are simply less selective for those fluctuations than are differentiators (Ratté et al., 2013), consistent with the observation that differentiators do not respond at all to slow-fluctuating stimuli whereas integrators do. In short, differences in subthreshold voltage trajectory and spike initiation confer differences in tuning that are directly reflected in the spike-triggered average and covariance (Aguera y Arcas and Fairhall, 2003; Gutkin et al., 2005; Fairhall et al., 2006; Ermentrout et al., 2007; Hong et al., 2007; Arthur et al., 2013). The present study extends the effort to link neuronal coding properties with spike initiation properties and, further, with biophysical properties.

Although we have emphasized the effects of different subthreshold currents, one must appreciate that thresholds arise from the nonlinear interaction between currents; therefore, subthreshold currents must be considered in the context of other currents expressed by the cell. To illustrate, consider that a weak slow-activating potassium current would pose little competition to a fast-activating sodium current that is particularly strong and would thus fail to confer differentiator traits to that neuron; however, if the sodium channel density was reduced, the same potassium channel density could prevent repetitive spiking and implement differentiator traits (Lundstrom et al., 2008). Likewise, cumulative sodium channel inactivation can reduce the availability of sodium channels (Fernandez et al., 2011) and increased leak conductance can increase perithreshold outward current (Zsiros and Hestrin, 2005; Prescott et al., 2006; Broicher et al., 2012), both with much the same effect as decreasing sodium channel density or increasing slow-activating potassium channel density. Indeed, simulations in Figure 4 demonstrated that sodium channel inactivation and potassium channel activation (with equivalent kinetics and voltage-dependency) can implement the same negative feedback and thereby confer the same differentiator traits. Lundstrom et al. (2009) modeled negative feedback as a combination of the two processes. But like canonical differentiators in the auditory midbrain (Reyes et al., 1996; Slee et al., 2005; Mathews et al., 2010; Higgs and Spain, 2011), certain pyramidal neurons in the neocortex (Guan et al., 2007), and dorsal root ganglion neurons (Ratté et al., 2014), differentiators in the spinal dorsal horn rely on a K_v_1 current for their response properties (Prescott et al., 2008a). Similarly, integrator traits can be conveyed by either inactivation of potassium current (Connor and Stevens, 1971; Cudmore et al., 2010; Barreiro et al., 2012) or by activation of sodium or calcium currents (Fricker and Miles, 2000; Gonzalez-Burgos and Barrionuevo, 2001; Vervaeke et al., 2006; Carter et al., 2012). The latter occur in tonic-spiking spinal neurons (Prescott and De Koninck, 2005; Prescott et al., 2008a).

To summarize, the spike initiation process is a critical component of neural coding. Spikes are not instantaneously initiated when voltage crosses a preordained threshold; on the contrary, spikes can be initiated through qualitatively distinct mechanisms that depend on the nonlinear interaction between contributing membrane currents. When slow-activating subthreshold current is inward, it will cooperate with the fast-activating current. Under those conditions, spike initiation relies on cumulative depolarization reaching a threshold—represented by the SNIC bifurcation—whereupon positive feedback activation of the slow inward current can guarantee activation of the fast current. When slow-activating subthreshold current is outward, it will compete with the fast-activating current. Under those conditions, spike initiation relies on depolarization activating the fast current quickly enough that the slow current cannot catch up, which is represented by the quasi-separatrix-crossing. In both cases, a stimulus must be of sufficient intensity to elicit a spike, but its interaction with the subthreshold current will control the preferred stimulus waveform: Integrators are tuned to slow depolarizing input—the sort of input that gives subthreshold inward current time to facilitate spiking—whereas differentiators are tuned to fast depolarizing input—the sort of input that gives subthreshold outward current too little time to prevent spiking. Thus, integration and differentiation arise from oppositely directed membrane currents and are implemented as part of the spike initiation process.

### Conflict of interest statement

The authors declare that the research was conducted in the absence of any commercial or financial relationships that could be construed as a potential conflict of interest.
